# Time-Resolved Network Entropy Analysis Identifies a CTNND1–VAV3 Subnetwork in Gefitinib-Resistant NSCLC

**DOI:** 10.3390/ijms27188409

**Published:** 2026-09-21

**Authors:** Dimitra C. Tsakona, Nikolaos A. Papanikolaou

**Affiliations:** 1 Inter-Faculty Program on Networks and Complexity, Aristotle University of Thessaloniki, 54124 Thessaloniki, Macedonia, Greece; dimitra.tsakona@outlook.com; 2 Department of Biology, Aristotle University of Thessaloniki, 54124 Thessaloniki, Macedonia, Greece; 3 Department of Basic Sciences, Touro College of Osteopathic Medicine, Touro University, New York, NY 10027, USA; 4 Laboratory of Biological Chemistry, Aristotle University of Thessaloniki School of Medicine, 54124 Thessaloniki, Macedonia, Greece; 5 Akesis Synergy Med, 49 Arkadiou Str., 56121 Thessaloniki, Macedonia, Greece

**Keywords:** gefitinib resistance, network entropy, protein interaction network, eigenvector centrality, CTNND1, VAV3, BIRC3, non-small cell lung cancer

## Abstract

Acquired resistance to epidermal growth factor receptor (EGFR) tyrosine kinase inhibitors remains the principal barrier to durable responses in non-small cell lung cancer (NSCLC), yet how the molecular interaction networks underlying this resistance reorganize over time is poorly understood. We reasoned that a systems-level signature of resistance should be visible in the way protein interaction networks remodel under sustained drug exposure, and therefore applied time-resolved network analysis to isogenic gefitinib-sensitive PC9 and gefitinib-resistant PC9R cells across 24 h of gefitinib treatment, computing network entropy and centrality measures for temporal protein interaction networks and interrogating co-expression-augmented networks for candidate resistance-associated bottleneck proteins. Network entropy rose in both phenotypes, indicating that entropic remodeling is a general response to EGFR pathway perturbation rather than a signature of resistance. In contrast, eigenvector entropy was higher in resistant cells at the earliest post-treatment time point, and resistant cells preserved giant-component connectivity and small-world topology early before fragmenting later. Temporal centrality analysis nominated BIRC3 as a resistant-cell-associated high-centrality node at 24 h, and co-expression analysis identified delta-catenin (CTNND1) as a high-betweenness bottleneck in the PC9R network, topologically bridging EGFR, VAV3, HIF3A, and NOTCH2. These findings nominate early post-treatment eigenvector entropy and a CTNND1-centered, EGFR-enriched subnetwork as candidate resistance-associated features that require validation in independent datasets and functional models.

## 1. Introduction

Non-small cell lung cancer (NSCLC) accounts for roughly 80–85% of lung cancer diagnoses and, in spite of major advances in targeted therapy and immunotherapy, remains a leading cause of cancer mortality [1,2]. Survival is strongly stage-dependent, with substantially better outcomes for localized than for metastatic disease. In molecularly selected NSCLC, activating mutations in the epidermal growth factor receptor (EGFR), most commonly exon 19 deletions and the exon 21 L858R substitution, confer sensitivity to EGFR tyrosine kinase inhibitors (EGFR-TKIs) such as gefitinib [3,4]. Even after pronounced initial responses, however, most tumors eventually acquire resistance through secondary EGFR mutations, bypass signaling, epithelial-to-mesenchymal transition (EMT), lineage plasticity, and broader transcriptomic reprogramming [5,6,7,8].

From a systems perspective, the functional state of a cell can be represented as a protein interaction network (PIN), in which nodes are proteins and edges are high-confidence protein–protein associations. Aberrant states, including drug resistance, can accordingly be conceptualized as topological and entropic changes in these networks [9,10,11,12,13,14,15,16,17]. Network entropy, which quantifies the diversification or randomness of local interaction patterns within a PIN, has emerged as a biologically meaningful measure of cellular state. West et al. demonstrated that PIN entropy is significantly elevated in cancer cells relative to matched normal tissue [18]; Banerji et al. showed that entropy scales with differentiation potential, with embryonic stem cells exhibiting the highest values [19]; and Breitkreutz et al. reported a correlation between PIN entropy and five-year survival in cancer patients [20]. Mechanistic support for these observations derives from the entropy-robustness theorem of Demetrius et al. [21], which formalizes a positive correlation between network robustness and entropy.

In spite of these advances, the time-resolved evolution of protein interaction network entropy under sustained EGFR inhibition, and its relationship to acquired drug resistance, remains incompletely characterized. We reasoned that gefitinib would drive broad network remodeling in both sensitive and resistant cells, but that the resistant-cell network might show candidate phenotype-associated differences in early entropy state, fragmentation dynamics, and bottleneck-node composition.

To test this, we analyzed publicly available time-course microarray data from isogenic gefitinib-sensitive PC9 and gefitinib-resistant PC9R NSCLC cells and reconstructed protein interaction networks from differentially expressed genes across five time points following gefitinib administration. This analysis revealed broad entropy elevation in both phenotypes, indicating that entropic remodeling is a general response to gefitinib exposure. In contrast, early post-treatment eigenvector entropy, delayed fragmentation, late BIRC3 centrality, and a CTNND1-centered EGFR–VAV3–HIF3A–NOTCH2 subnetwork emerged as candidate resistant-cell-associated features. Because the study is entirely computational and based on a single isogenic cell-line system, these findings are hypothesis-generating and require experimental and clinical validation.

## 2. Results

### 2.1. Differential Gene Expression After Gefitinib Administration

Gefitinib-sensitive PC9 and gefitinib-resistant PC9R cells both displayed robust, bidirectional differential gene expression at each time point (Figures S1 and S2). Volcano plots confirmed substantial differential-expression signals, and median-centered boxplot distributions confirmed inter-sample normalization quality. Limma-based filtering (adj-*p* < 0.05) yielded variable numbers of significant DEGs at individual time points in both cell lines. Across the full 24 h treatment window used for co-expression PIN construction, PC9 cells yielded 19,995 significant DEGs and PC9R cells 16,776, providing a rich input for the downstream network analysis.

### 2.2. Global Topology of Temporal PINs Is Sparse, Assortative, and Compatible with Heavy-Tailed Organization

The temporal protein interaction networks of both cell lines were sparse (density ≤ 0.022 at all time points), disconnected (19–50 components), and displayed heavy-tailed degree distributions compatible with power-law behavior at most time points (Kolmogorov–Smirnov *p* > 0.05; scaling exponents α ≈ 1.7–3.8). These properties are consistent with hub-dominated organization without requiring a strict scale-free interpretation. Notably, all networks were assortative at every time point, indicating that highly connected proteins preferentially interacted with other highly connected proteins. Selected global properties are reported in Table 1 and Table 2, and the complete set is provided in Tables S1 and S2.

### 2.3. Progressive Network Fragmentation Under Sustained Gefitinib Treatment

**Figure 1:**
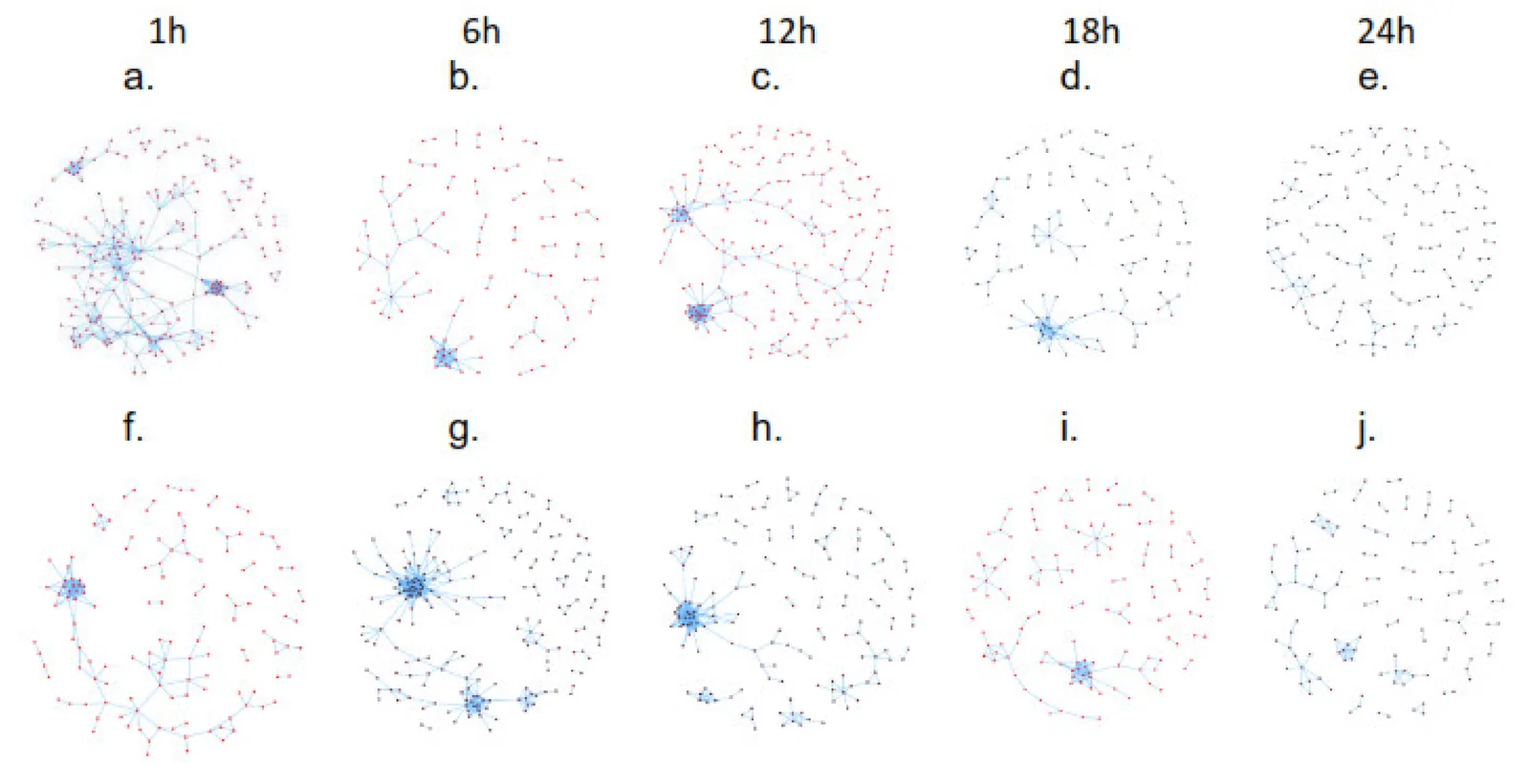
Protein interaction networks of differentially expressed genes in gefitinib-sensitive PC9 cells (**a**–**e**) and gefitinib-resistant PC9R cells (**f**–**j**) at five time points (1 h, 6 h, 12 h, 18 h, and 24 h) after gefitinib administration. Red nodes represent proteins encoded by differentially expressed genes, and light-blue edges represent STRING-derived protein–protein associations. The progressive reduction in the giant component reflects network rewiring under sustained EGFR pathway suppression.

PC9 PINs showed a progressive reduction in order, size, and giant component (GC) across the 24 h treatment period (Table 1 and Table 2). In PC9R cells, by contrast, the network parameters first expanded at 6 h (order 138 → 218; edges 200 → 543; GC 52% → 59%) and only then fragmented progressively from 12 h onward, suggesting an early compensatory rewiring response that is absent from sensitive cells. In PC9 cells, the GC declined from 73% at 1 h to 12% at 24 h; in PC9R cells, from 52% to 14% (Figure 1). This fragmentation reflects a loss of connectivity among the most drug-responsive proteins under sustained EGFR pathway suppression. Although both cell lines fragmented, the sensitive PC9 network fragmented more severely by 24 h, whereas the resistant PC9R network retained more connectivity through 6 h (GC = 59%). This pattern is consistent with transient preservation of network connectivity in PC9R cells, although it does not by itself establish a causal resistance mechanism.

### 2.4. Network Entropy Trajectories Under Gefitinib Treatment

**Figure 2:**
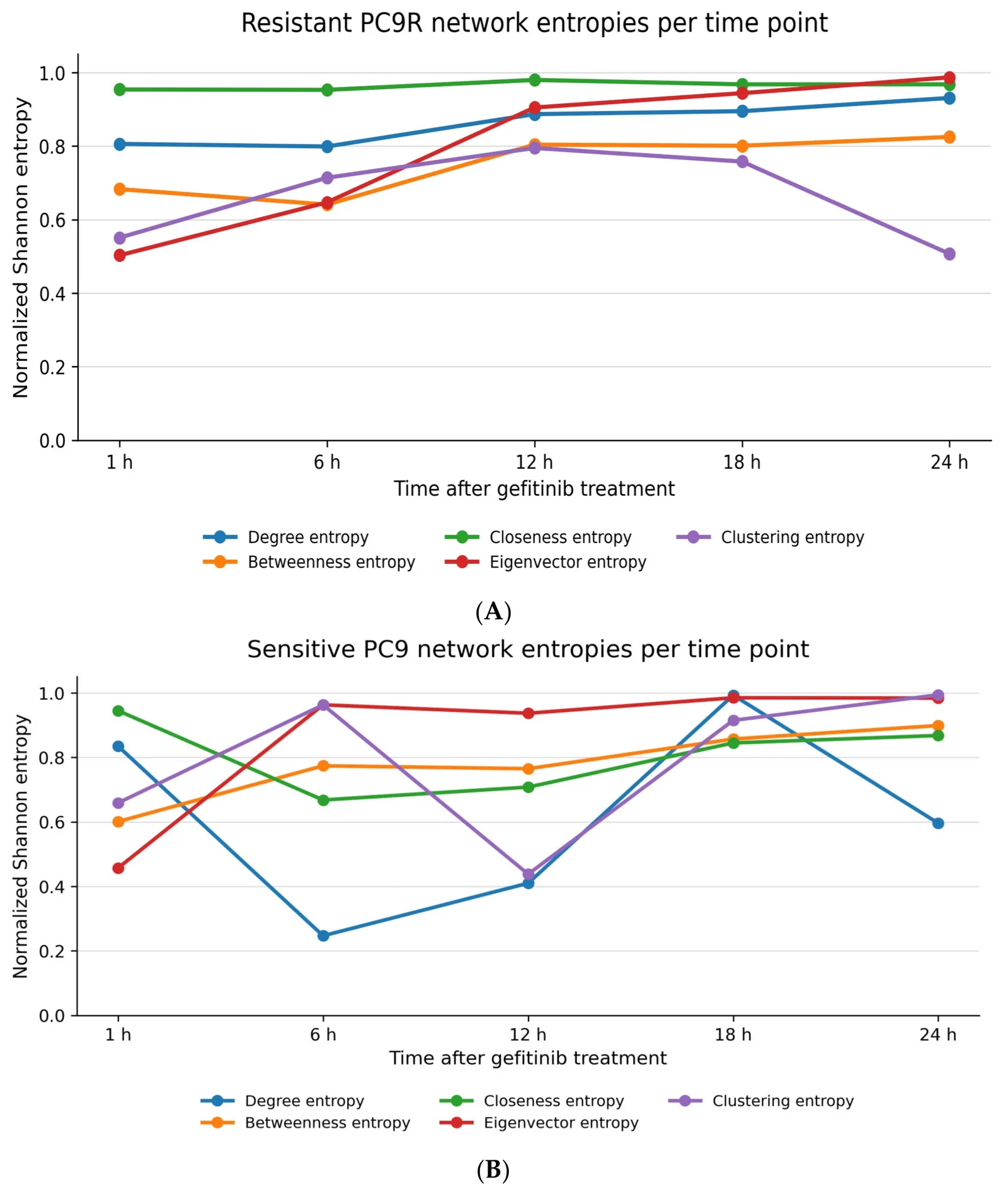
Network entropy trajectories of node-property distributions in gefitinib-resistant PC9R cells (**A**) and gefitinib-sensitive PC9 cells (**B**) at five time points under gefitinib treatment. Most entropy measures show an overall upward tendency over the 24 h course in both phenotypes, despite non-monotonic fluctuations in several individual metrics.

Entropy values showed an overall upward tendency over the 24 h treatment period in both PC9 and PC9R cells, although individual metrics fluctuated non-monotonically at intermediate time points (Figure 2). When the five entropy metrics were summarized by their median at each time point, median entropy increased from 0.659 at 1 h to 0.899 at 24 h in PC9 cells and from 0.683 at 1 h to 0.931 at 24 h in PC9R cells. Linear trend analysis of median entropy returned a positive slope in both phenotypes, with stronger temporal consistency in PC9R than in PC9 cells. Across all 25 paired entropy values, however, the overall PC9R–PC9 difference was small and not statistically significant in exploratory paired analysis. Thus, our data support the conclusion that entropy elevation is a general response to gefitinib exposure rather than a resistance-associated signature.

Eigenvector entropy, on the other hand, showed a modest early phenotype difference: at the earliest measured post-treatment time point, PC9R cells had higher eigenvector entropy than PC9 cells (0.503 vs. 0.457). By 24 h, the two phenotypes converged to nearly identical high values (0.987 in PC9R and 0.984 in PC9). Because this comparison rests on a single network per phenotype at each time point, it should not be read as a validated biomarker difference; rather, early eigenvector entropy is best regarded as a candidate systems-level feature of the resistant state that requires validation in independent datasets and, ideally, in pretreatment transcriptomic or proteomic profiles linked to EGFR-TKI response. Accordingly, we use this observation only to motivate further validation studies and not as inferential evidence that eigenvector entropy is a general biomarker of gefitinib resistance.

### 2.5. Progressive Fragmentation and Loss of Small-World Topology

Both phenotypes lost giant-component connectivity over the 24 h period, but the temporal pattern differed between PC9 and PC9R cells. In PC9 cells, the giant component declined from 73% at 1 h to 12% at 24 h, along a non-monotonic trajectory marked by partial recovery at 12 h. In PC9R cells, the giant component initially expanded from 52% at 1 h to 59% at 6 h, followed by a more monotonic decline to 14% at 24 h. Exploratory trend analysis was consistent with stronger temporal monotonicity of fragmentation in PC9R cells, whereas PC9 showed sharper early fragmentation but greater intermediate fluctuation. The resistant-cell network, therefore, does not simply fragment less; rather, it preserves or even expands connectivity early and collapses later.

Small-world topology, likewise, was lost earlier in PC9 than in PC9R cells. PC9 retained a small-world index greater than 1 only at 1 h, whereas PC9R retained small-world topology at both 1 h and 6 h before falling below threshold from 12 h onward. This supports a conservative interpretation in which resistant cells transiently preserve clustered, short-path organization under early gefitinib exposure. Because the networks become highly fragmented at later time points, centrality and entropy values derived from the full graph should be interpreted with caution. Differences in entropy and centrality may partly reflect network size and fragmentation rather than biological rewiring alone. A giant-component-restricted reanalysis would therefore be required to determine whether the reported trends are robust to fragmentation.

### 2.6. Key Hub and Bottleneck Proteins Nominate Candidate Phenotype-Associated Regulators

Proteins with maximum centrality were identified at each time point in both cell lines (Table 3). In sensitive PC9 cells, prominent hubs included CREBBP (1 h; chromatin remodeling), CDK1 (6 h; G1/S transition), MYC (12 h; proliferation and apoptosis), CDC6 (18 h; DNA replication licensing), and RRM2 (24 h; nucleotide synthesis). The progressive shift from chromatin-remodeling factors to DNA-synthesis machinery is consistent with an ordered transcriptional response to EGFR inhibition in sensitive cells.

In resistant PC9R cells, the centrality landscape differed substantially. NOP56 (1–6 h; rRNA processing), CDC6 (12 h), and EXO1 (18 h; DNA repair and replication) dominated at early-to-mid time points. Notably, at 24 h, BIRC3, a Baculoviral IAP Repeat Containing 3 protein and inhibitor-of-apoptosis family member, became the highest-ranking resistant-cell node by degree, betweenness, and closeness centrality, whereas CASP10 showed the highest eigenvector centrality. The concurrent prominence of BIRC3 and CASP10 points to a late enrichment of apoptosis-regulatory nodes in the resistant network. BIRC3 has been associated with poor overall survival in NSCLC and with chemoresistance in colorectal cancer [22,23,24]; its emergence as a high-centrality PC9R node at 24 h therefore nominates it as a candidate late-stage apoptosis-associated node in the resistant-cell network.

### 2.7. Co-Expression Network Analysis Nominates Candidate Resistance-Associated Bottleneck Nodes

To place these observations in a broader regulatory context, we constructed co-expression-augmented PINs for PC9 and PC9R cells. Their global topological properties were broadly comparable (Table 4; Figure S3): both exhibited small-world organization (PC9: 3.180; PC9R: 2.632), positive assortativity, and low density. This global equivalence is important because it renders the two networks structurally matched at a systems level and thereby makes local centrality differences more meaningful.

**Figure 3:**
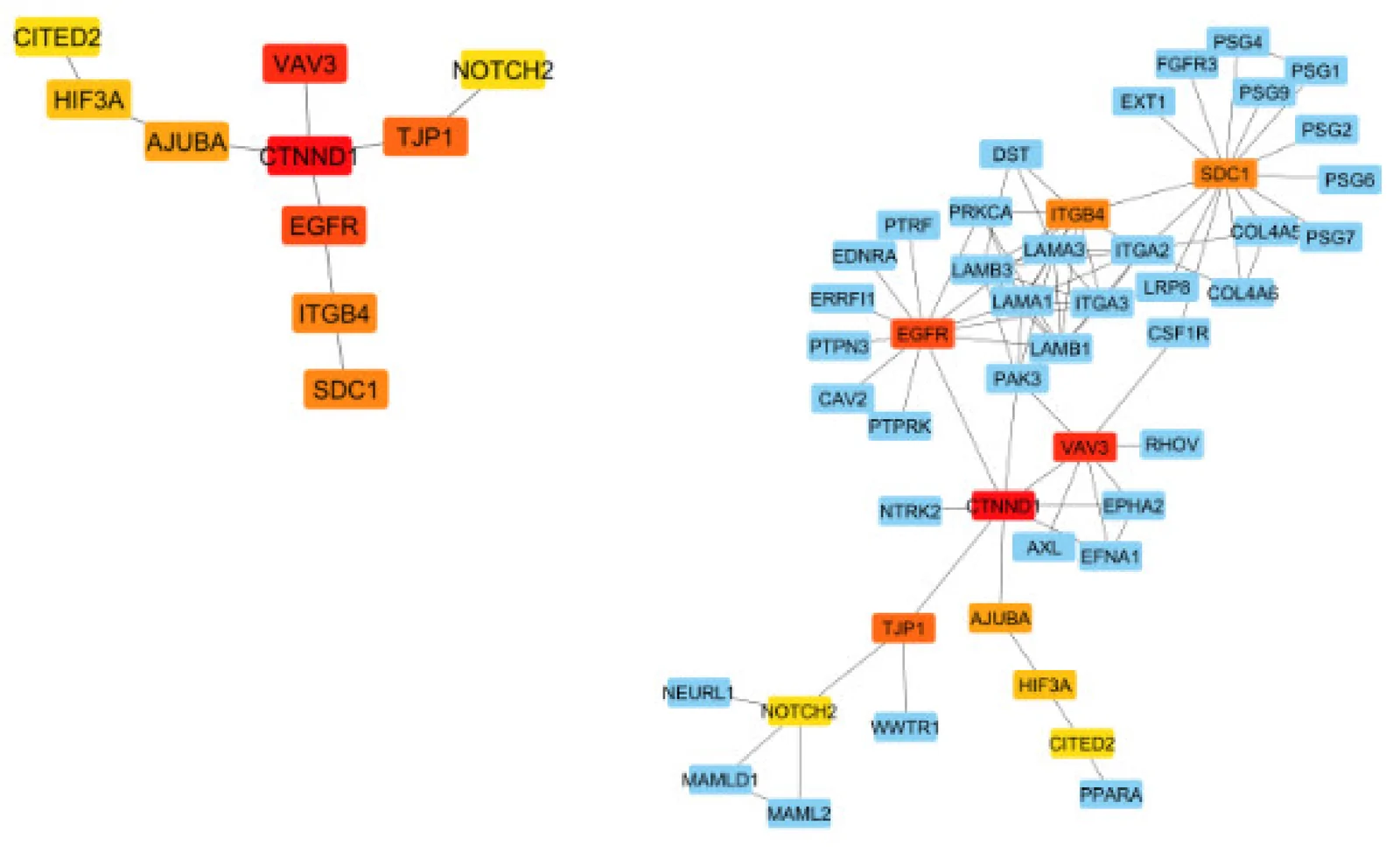
Candidate resistance-associated co-expression subnetwork in gefitinib-resistant PC9R cells. The top-10 betweenness-centrality (bottleneck) nodes of the PC9R-resistant network, with CTNND1 (δ-catenin) as a high-betweenness bridge node, and its first-order interactors revealing EGFR–CTNND1–VAV3–NOTCH2–HIF3A connectivity. Nodes not present among the dominant bottlenecks of the gefitinib-sensitive PC9 network include VAV3, HIF3A, NOTCH2, CITED2, and RhoV. Node colors indicate betweenness-centrality ranking among the top-10 bottleneck nodes (red, highest ranked, through orange to yellow, lower ranked); light-blue nodes represent first-order interactors.

In spite of these comparable global properties, the composition of the top-10 betweenness-centrality (bottleneck) nodes differed markedly. Within this dataset, the PC9R network contained VAV3, HIF3A, NOTCH2, and CITED2 among its top-10 bottleneck nodes, whereas these proteins were not among the top-10 bottlenecks of the PC9 network (Figure 3). Of particular interest, delta-catenin (CTNND1) was the top betweenness-centrality node in the PC9R co-expression network, acting topologically as a bridge connecting EGFR, VAV3, NOTCH2, and HIF3A within a single subnetwork. RhoV also appeared as a VAV3 interactor in the PC9R network. Taken together, these findings nominate a candidate EGFR-enriched, resistance-associated subnetwork (Figure 3) that forms the basis of the hypothesis-generating model developed in the Discussion.

Because 300 of the 500 seed genes were selected from EGFR co-expression data in COXPRESdb, the co-expression-augmented network may be enriched for EGFR-associated connectivity; this design was useful for interrogating EGFR-centered resistance biology, but it may also favor the emergence of EGFR-linked subnetworks. Therefore, the CTNND1–VAV3–HIF3A–NOTCH2 module should be interpreted as a candidate EGFR-enriched, resistance-associated structure rather than definitive evidence of resistance specificity.

## 3. Discussion

### 3.1. Overview of Main Findings

This study provides a time-resolved, systems-level analysis of protein interaction network entropy and topology in isogenic gefitinib-sensitive and gefitinib-resistant NSCLC cells. Our central conclusion is that network entropy should not be read as a simple monotonic marker of drug resistance. Entropy increased broadly in both phenotypes under gefitinib treatment, indicating that entropic remodeling is a general response to EGFR pathway perturbation. Candidate resistant-cell-associated information emerged instead from early post-treatment eigenvector entropy, delayed early fragmentation, late enrichment of apoptosis-regulatory centrality nodes, and the composition of a CTNND1-centered co-expression subnetwork observed in the PC9R network. These features should be interpreted as hypothesis-generating network observations rather than validated resistance mechanisms.

### 3.2. Network Entropy Increase as a Universal Response to Gefitinib

The universal entropy increase observed in both sensitive and resistant cells is grounded in two complementary frameworks. First, the entropy-robustness theorem of Demetrius et al. predicts that external perturbations drive biological networks toward higher-entropy states as they engage compensatory pathways [21]. Second, the progressive loss of small-world topology in both phenotypes reflects the dismantling of EGFR-centered signaling hubs: gefitinib inhibits EGFR autophosphorylation at the tyrosine kinase domain and thereby fragments the tightly clustered signaling modules organized around EGFR, the central hub of that architecture. Disrupting such hubs in scale-free networks is well known to degrade small-world properties by fragmenting hub-mediated inter-cluster communication [25].

That entropy increases universally in both phenotypes carries an important nuance: resistance cannot be read off from entropy trajectories alone. Rather, in this dataset, candidate phenotype-associated information is found in the earliest measured post-treatment state and in specific topological differences, including centrality landscape and co-expression subnetwork composition. This distinction is critical both for the correct interpretation of network entropy as a resistance descriptor and for the design of future studies.

### 3.3. Early Eigenvector Entropy as a Hypothesis-Generating Network Feature

Eigenvector entropy captures the extent to which regulatory influence is distributed across all network nodes rather than concentrated in a few central hubs; a higher value therefore reflects a more democratically connected network and greater robustness to targeted perturbation [19,21]. The eigenvector entropy difference at 1 h was modest (0.503 in PC9R vs. 0.457 in PC9) and was derived from one network per phenotype. We therefore do not interpret this observation as a validated biomarker or as inferential evidence of a general resistance signature. Rather, it suggests a testable hypothesis: future studies should determine whether eigenvector entropy calculated from true pretreatment tumor transcriptomes or proteomes is associated with EGFR-TKI response in independent cohorts.

### 3.4. Candidate Resistance-Associated Hub Proteins: BIRC3 and UBE2D1

The emergence of BIRC3 as the high-centrality PC9R node by degree, betweenness, and closeness centrality at 24 h is among the most biologically compelling findings of this study. BIRC3 belongs to the inhibitor of apoptosis (IAP) family and directly suppresses activation of caspases-3, -7, and -9 through its BIR domains [26,27]. In NSCLC, higher BIRC3 expression has been independently associated with poorer overall survival [22], and in colorectal and hepatocellular cancers, its upregulation has been linked to chemoresistance through inhibition of 5-FU-induced apoptosis, promotion of EMT, and facilitation of metastasis [23,24,28]. Our network analysis positions BIRC3 as a late-stage (24 h) high-centrality apoptosis-associated node in the PC9R response to gefitinib. This raises the hypothesis that BIRC3 may contribute to apoptosis buffering in resistant cells, but this possibility requires functional testing. BIRC3 knockdown or pharmacologic inhibition in PC9R cells under gefitinib treatment would provide a direct test of this hypothesis.

At 6 h in resistant cells, UBE2D1 held the highest betweenness centrality. UBE2D1 participates in the ubiquitination and proteasomal degradation of p53 and HIF1α through interaction with E3 ubiquitin-protein ligases, including MDM2 [29]. As an early-time-point bottleneck observed in the PC9R network, UBE2D1 may represent a candidate ubiquitin-related node linked to hypoxia/HIF-associated signaling, but its functional role in gefitinib resistance remains to be tested.

### 3.5. A Hypothesis-Generating CTNND1–VAV3–HIF3A–NOTCH2 Model Derived from Network Topology

A central network-derived observation of this study is that CTNND1 appears as a high-betweenness bridge in the PC9R co-expression-augmented network, linking EGFR, VAV3, HIF3A, and NOTCH2. This observation does not establish regulatory directionality or causality. Instead, it identifies a topological structure that can be integrated with prior biological literature to generate an experimentally testable model.

The mechanistic plausibility of this subnetwork rests on three converging lines of evidence. First, EGFR phosphorylates delta-catenin, enhancing EGFR signaling in a positive feedback loop [30]. Second, hypoxia-associated HIF signaling has been reported to induce CTNND1 expression [31], and delta-catenin, in turn, stabilizes β-catenin by disrupting the destruction complex, thereby activating Wnt signaling. Third, delta-catenin regulates Rho-family GTPase activity, inhibiting RhoA and activating Rac1/Cdc42, and thereby modulating actin cytoskeletal dynamics and cell motility [32,33]. VAV3, identified here as a candidate resistance-associated bottleneck in the PC9R network, is a guanine nucleotide exchange factor (GEF) that activates Rho-family GTPases by catalyzing GDP-to-GTP exchange [34]; it has been functionally linked to endocrine-therapy resistance in breast cancer [35] and to drug resistance in gastric cancer through MAPK/PI3K/AKT modulation [36]. Its role in EGFR-TKI resistance in NSCLC is, however, unexplored, and our network finding provides topological evidence consistent with VAV3 involvement in the resistant phenotype.

NOTCH2, for its part, has a well-established role in NSCLC survival, stemness, and EGFR-TKI resistance [37,38,39,40]. Critically, Notch signaling is required to maintain the undifferentiated cell state under hypoxia [41], and hypoxia has itself been linked to gefitinib resistance through upregulation of FGFR1/MAPK [42] and TGFα/EGFR axes [43]. HIF3A, finally, has been detected at aberrantly elevated levels in the plasma and tumor tissue of NSCLC patients [44].

**Figure 4:**
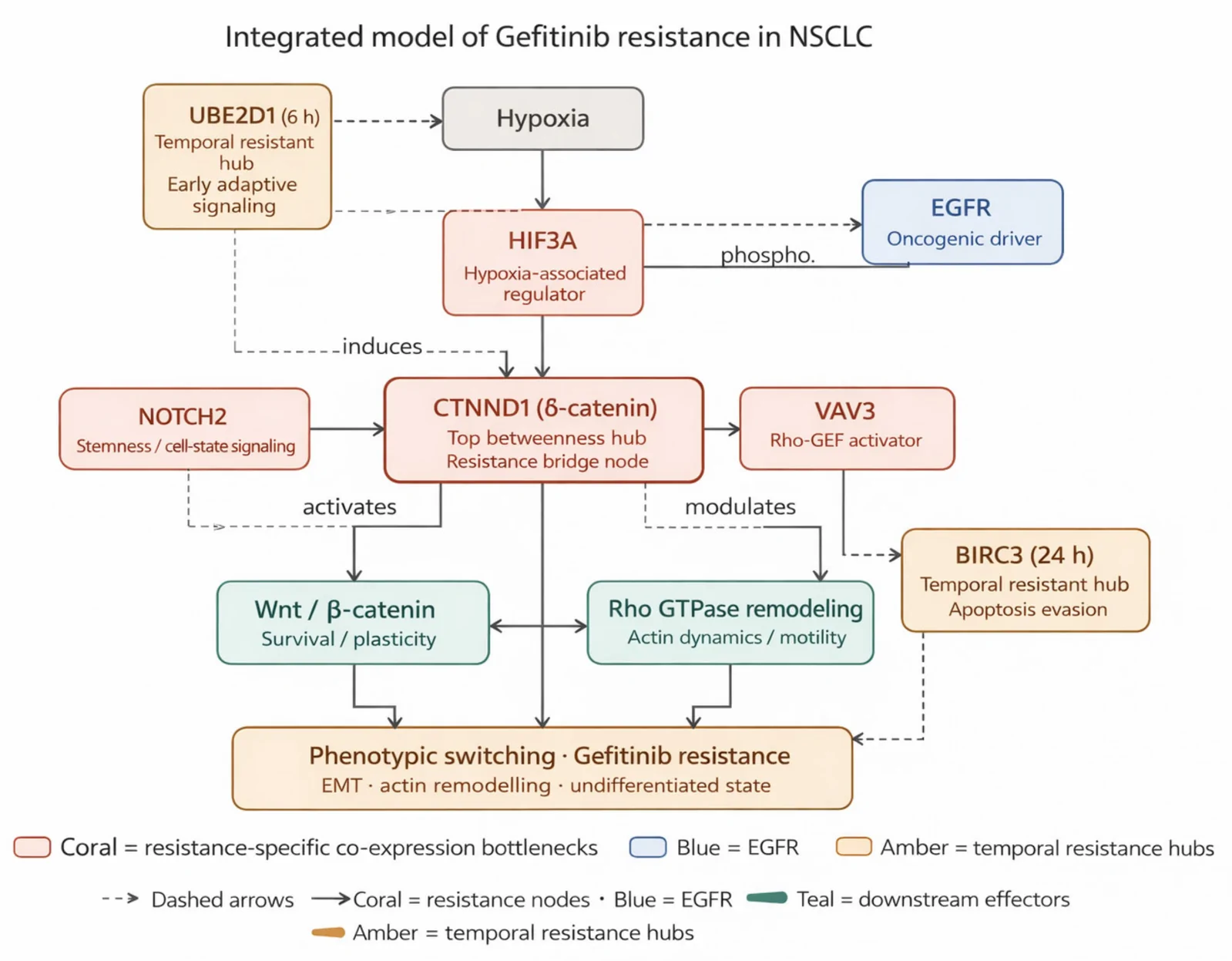
Hypothesis-generating two-layer model derived from network topology and prior literature in gefitinib-resistant NSCLC. A co-expression-derived resistance core centered on CTNND1 (δ-catenin) links HIF3A, NOTCH2, EGFR, and VAV3 to downstream survival signaling and cytoskeletal remodeling. UBE2D1 and BIRC3 are shown separately as early and late temporal resistance-associated hubs, respectively; their functional roles in gefitinib resistance remain to be tested. Solid arrows denote evidence-supported activating relationships; dashed arrows denote temporal or hypothesized links. The model summarizes experimentally testable hypotheses and should not be interpreted as evidence of causal regulation by network topology alone.

Integrating these observations, we propose a hypothesis-generating model in which hypoxia-associated signaling, EGFR-linked delta-catenin regulation, Notch signaling, and VAV3/Rho-GTPase biology may converge within the PC9R network (Figure 4). The resistant network topology is compatible with, but does not prove, a model in which CTNND1 and VAV3 participate in Rho-GTPase-linked cytoskeletal remodeling, epithelial-to-mesenchymal transition, and survival signaling. This model is experimentally testable: CTNND1 or VAV3 perturbation in PC9R cells under gefitinib treatment could determine whether these nodes influence Rho-GTPase activity, apoptosis, and gefitinib sensitivity; patient-cohort analyses could determine whether HIF3A, CTNND1, VAV3, or NOTCH2 expression correlates with EGFR-TKI response.

### 3.6. Network Fragmentation and the Survival of the Sparsest Principle

The progressive reduction in giant-component size indicates fragmentation of the DEG-derived PINs under sustained gefitinib exposure. This may reflect biological rewiring, but it may also affect entropy and centrality estimates directly [45]. We therefore interpret fragmentation as both a biological observation and a methodological factor. Because network size, edge number, and giant-component structure differ across time points, whole-network entropy and centrality values should be interpreted cautiously until confirmed by giant-component-restricted analysis [46].

### 3.7. Limitations and Future Directions

Several limitations must be acknowledged. First, the use of the top 1000 differentially expressed genes per phenotype and time point was chosen to generate networks of manageable and broadly comparable size, but this cutoff is methodological rather than biologically intrinsic. We did not reconstruct temporal PINs at alternative DEG thresholds, such as the top 500, top 1500, or all genes passing adj-*p* < 0.05. Consequently, the robustness of the reported entropy trajectories, giant-component dynamics, small-world remodeling, and centrality rankings to DEG-threshold selection remains untested. These findings should therefore not be interpreted as threshold-independent. Future work should perform formal sensitivity analyses across multiple cutoffs and determine whether the main candidate features—early eigenvector entropy differences, delayed small-world loss in PC9R cells, late BIRC3 centrality, and CTNND1–VAV3 subnetwork structure—remain stable.

Second, the entropy estimates depend on discretization of node-property distributions before Shannon entropy calculation. Although the same computational workflow was applied across networks, we did not conduct a formal sensitivity analysis comparing alternative entropy-binning strategies. Therefore, the reported entropy trajectories may partly depend on discretization choices, including bin number, bin width, and the use of fixed-width versus quantile-based binning. Future work should explicitly compare alternative binning schemes and determine whether the observed entropy trends remain stable.

Third, the co-expression-augmented network was assembled from three heterogeneous sources: GEO-derived DEGs, GSEA leading-edge genes, and COXPRESdb-derived EGFR co-expressed genes. Because 300 of the 500 seed genes were selected from EGFR co-expression data, the resulting network may be enriched for EGFR-associated connectivity; this design was appropriate for exploring EGFR-centered resistance biology, but it may also favor the emergence of EGFR-linked subnetworks. Therefore, the CTNND1–VAV3–HIF3A–NOTCH2 module should be interpreted as a candidate EGFR-enriched, resistance-associated structure rather than definitive evidence of resistance specificity. Future work should repeat the co-expression network construction after excluding or varying the COXPRESdb-derived component.

A further caveat concerns the underlying interaction database. Well-studied proteins such as EGFR, MYC, and the NOTCH family carry far more annotated STRING associations than understudied proteins of equal biological importance, and this annotation bias inflates the centrality of canonical cancer genes in any STRING-derived network, so that analyses of this kind may recapitulate known biology while underweighting genuinely novel candidates. Replication using interaction databases with different annotation profiles (e.g., HuRI, BioPlex) would strengthen the findings. In the same spirit, the candidate resistance-associated features identified here—eigenvector entropy, BIRC3 centrality, and the CTNND1–VAV3 subnetwork—could in principle be tested in independent transcriptomic cohorts with linked EGFR-TKI response data. We did not perform this validation here, and its absence is a substantive limitation. Therefore, eigenvector entropy, BIRC3 centrality, and the CTNND1–VAV3 subnetwork should be considered candidate findings from one isogenic model system until reproduced in independent EGFR-mutant or EGFR-TKI-response datasets.

Finally, network topology alone cannot establish whether CTNND1 centrality, BIRC3 hub status, or elevated eigenvector entropy are causal drivers of the resistant phenotype or downstream consequences of an upstream resistance event, such as a secondary EGFR mutation. Functional experiments are required to distinguish causation from correlation in network space.

## 4. Materials and Methods

### 4.1. Dataset

Gene expression data from gefitinib-sensitive (PC9) and gefitinib-resistant (PC9R) NSCLC cells were retrieved from the NCBI Gene Expression Omnibus (accession GSE34228). This publicly available dataset, generated by Minakata et al. [43], comprises Affymetrix Human Genome U133 Plus 2.0 microarray measurements at five time points (1 h, 6 h, 12 h, 18 h, 24 h) following gefitinib treatment, together with paired untreated controls for both cell lines. All analyses were performed on publicly available, fully anonymized data; no new patient-derived or animal samples were used.

### 4.2. Differential Expression Analysis

Differential expression between gefitinib-treated and untreated samples was assessed with GEO2R at https://www.ncbi.nlm.nih.gov/geo/geo2r/ (accessed on 10 September 2026), the NCBI web interface that executes the limma Bioconductor package on the GEO server. Default GEO2R settings were applied, including log2-transformation of expression values prior to model fitting and Benjamini–Hochberg false-discovery-rate adjustment of *p*-values. For each gene at each time point, we obtained the log2 fold change (log2FC) and adjusted *p*-value (adj-*p*), and genes with adj-*p* < 0.05 were considered significantly differentially expressed (DEGs). For temporal protein interaction network (PIN) construction, the top 1000 DEGs ranked by |log2FC| per cell line per time point were retained, whereas for co-expression-augmented PIN construction all significant DEGs across the full 24 h treatment window were used (PC9: 19,995; PC9R: 16,776). This threshold was selected to generate networks of manageable and broadly comparable size across phenotype/time-point contrasts; it was not intended to represent a biologically defined cutoff. Identical contrasts can be reproduced by selecting the corresponding treated and untreated sample groups in the GEO2R interface.

### 4.3. Construction of Temporal Protein Interaction Networks

For each combination of cell line and time point, the corresponding top-1000 DEG list was submitted to the STRING database, version 11 at https://string-db.org/ (accessed on 10 September 2026) [47], through the multiple-protein search interface with the organism parameter set to *Homo sapiens*. Active interaction sources were restricted to “experiments” and “databases”; co-expression, text mining, gene neighborhood, gene fusion, and co-occurrence were disabled. The minimum required interaction score was left at the STRING default of medium confidence (0.400), and the resulting networks were exported in TSV format. This procedure yielded ten temporal PINs (5 time points × 2 cell lines).

### 4.4. Construction of Co-Expression-Augmented Protein Interaction Networks

To broaden the regulatory context beyond directly differentially expressed transcripts, co-expression-augmented PINs were constructed separately for each cell line. For each cell line, a 500-gene seed list was assembled from three sources: (i) the top 100 DEGs ranked by |log2FC| across the full 24 h treatment window, obtained from GEO2R as described above; (ii) 100 leading-edge genes derived from significantly enriched Hallmark gene sets (MSigDB v7.4) identified by gene set enrichment analysis (GSEA v4.1, Broad Institute desktop application) [48], run on the same dataset with default parameters (1000 gene-set permutations, weighted enrichment statistic, gene-set size 15–500); and (iii) 300 EGFR co-expressed genes retrieved from COXPRESdb v7 at https://coxpresdb.jp/ (accessed on 10 September 2026) [49] using EGFR as the query protein with default settings on the Hsa-r.c2-0 reference platform. The combined 500-gene seed list per cell line was then submitted to STRING v11 with the parameters described above to generate the co-expression-augmented PIN. Because the COXPRESdb component was seeded on EGFR, this network-construction strategy may enrich for EGFR-associated interactions and was interpreted accordingly as an EGFR-centered exploratory analysis.

### 4.5. Network Analysis and Visualization

All network analyses and visualizations were performed in Cytoscape v3.8.2 [50] using the following plugins: (i) NetworkAnalyzer (built-in) [51] for global topological properties—order, size, density, number of connected components, giant-component (GC) order and size, diameter, average shortest-path length, global and local clustering coefficients (Watts–Strogatz transitivity), and degree assortativity; (ii) CytoHubba [52] for ranking nodes by degree, betweenness, closeness, and eigenvector centrality and for extracting the top-10 betweenness-centrality (bottleneck) subnetworks together with their first-order interactors (Figure 3); and (iii) CytoNCA [53] for centralization measures and cross-validation of centrality scores. Erdős–Rényi reference graphs matched on node and edge counts were generated within Cytoscape to normalize the small-world calculations.

The small-world index σ was computed as σ = (C/C_rand)/(L/L_rand), where C and L are the observed clustering coefficient and average path length, and C_rand and L_rand are the corresponding values for a size-matched Erdős–Rényi random graph [54]; networks with σ > 1 were classified as small-world.

Degree distributions were assessed for compatibility with power-law behavior using the maximum-likelihood framework of Clauset, Shalizi, and Newman [55], which returns the scaling exponent α, the lower-bound parameter x_min, and a Kolmogorov–Smirnov goodness-of-fit *p*-value; *p* > 0.05 was taken as compatibility with a power-law tail. This compatibility test does not establish a power-law as the best-fitting model relative to alternative heavy-tailed distributions (e.g., lognormal, exponential, or stretched-exponential) and is therefore reported descriptively, to characterize heavy-tailed organization rather than as evidence of strict scale-free structure.

Five entropy metrics were computed for the temporal PINs: degree entropy, betweenness entropy, closeness entropy, eigenvector entropy, and clustering entropy. For a discrete probability distribution {p_1_, p_2_, …, p_m}, with Σp_i = 1, Shannon entropy was calculated as H = −Σp_i ln(p_i), and normalized as H_norm = H/ln(m), yielding values between 0 and 1. For the co-expression-augmented PINs, PageRank entropy was also computed using the same Shannon-entropy normalization. Node-property distributions exported from Cytoscape were discretized before entropy calculation in Microsoft Excel. Because entropy estimates can depend on discretization choices, including bin number and bin width, the absence of a formal binning-sensitivity analysis is a limitation of the present study. The spreadsheet template used for entropy calculation is included in the Supplementary Materials.

### 4.6. Software, Tool Versions, and Reproducibility

All software, web platforms, and database versions used in this study, together with access dates and parameter settings, are listed in Table S4. Intermediate data files—DEG lists, GSEA leading-edge gene sets, COXPRESdb seed lists, STRING-exported edge files for all twelve networks (10 temporal PINs plus 2 co-expression-augmented PINs), Cytoscape session files (.cys), and the Excel entropy-computation template—are available upon reasonable request and will be deposited in a public repository where permitted.

### 4.7. Exploratory Statistical Analysis of Network-Level Metrics

Because each phenotype–time-point combination generated a single protein interaction network, statistical analyses of network-level metrics were treated as exploratory and descriptive rather than as inferential tests of biological replication. For each topological or entropy metric reported across the five time points, PC9R–PC9 differences were calculated at matched time points, and exact paired sign-flip permutation tests were used to evaluate whether the mean paired difference across time points differed from zero; Wilcoxon signed-rank tests were also calculated where applicable. Temporal trends were assessed by linear regression of each metric against treatment time and by Spearman rank correlation. For entropy-trajectory summaries, the median and mean of the five entropy metrics—degree, betweenness, closeness, eigenvector, and clustering entropy—were calculated at each time point for each phenotype. Because the five time points are temporally ordered and not independent biological replicates, *p*-values from these analyses were interpreted only as descriptive measures of temporal consistency. Accordingly, these analyses were not used to claim statistically validated candidate phenotype-associated effects; they were used only to summarize temporal patterns and guide hypothesis generation.

## 5. Conclusions

In summary, time-resolved network entropy analysis distinguishes broad gefitinib-induced remodeling from candidate resistant-cell-associated topological features in isogenic NSCLC cells. The CTNND1–VAV3–HIF3A–NOTCH2 subnetwork and the late-stage BIRC3 hub provide testable starting points for experimental dissection of EGFR-TKI resistance. Because the study is computational and based on one isogenic model system, these features should be viewed as hypothesis-generating until confirmed by independent cohort validation and functional perturbation studies.

## Figures and Tables

**Table 1:** Global network properties of gefitinib-sensitive PC9 temporal PINs.

Measure	1 h	6 h	12 h	18 h	24 h
Order (nodes)	221	118	206	124	164
Size (edges)	478	140	358	150	128
No. of components	19	31	32	30	50
GC order	162	30	124	35	20
GC size	404	30	304	82	24
GC (% of total)	73%	25%	60%	28%	12%
Avg. path length	4.296	3.331	8.616	3.267	2.609
Graph density	0.019	0.020	0.016	0.019	0.009
Assortativity	0.441	0.695	0.597	0.562	0.270
Small-world index	5.157 ^1^	0.048	0.626	0.578	0.231
Degree entropy	0.835	0.247	0.410	0.993	0.596
Betweenness entropy	0.601	0.774	0.765	0.857	0.899
Closeness entropy	0.945	0.668	0.708	0.845	0.868
Eigenvector entropy	0.457	0.963	0.937	0.985	0.984
Clustering entropy	0.659	0.963	0.438	0.915	0.994

Abbreviations: PINs, protein interaction networks; GC, giant component; No., number; Avg., average; h, hours. PC9 denotes the gefitinib-sensitive cell line. ^1^ Small-world index > 1 indicates networks retaining small-world topology. Entropy values are normalized Shannon entropy (range 0–1). Complete properties are provided in Table S1.

**Table 2:** Global network properties of gefitinib-resistant PC9R temporal PINs.

Measure	1 h	6 h	12 h	18 h	24 h
Order (nodes)	138	218	168	134	128
Size (edges)	200	543	308	158	119
No. of components	23	32	34	31	39
GC order	72	129	53	28	18
GC size	151	475	201	73	18
GC (% of total)	52%	59%	32%	21%	14%
Avg. path length	6.215	6.337	3.488	2.952	2.672
Graph density	0.021	0.022	0.021	0.017	0.014
Assortativity	0.625	0.408	0.518	0.587	0.502
Small-world index	2.030 ^1^	1.703 ^1^	0.981	0.906	0.337
Degree entropy	0.806	0.799	0.887	0.895	0.931
Betweenness entropy	0.683	0.641	0.804	0.801	0.825
Closeness entropy	0.954	0.953	0.980	0.968	0.968
Eigenvector entropy	0.503	0.647	0.905	0.944	0.987
Clustering entropy	0.551	0.714	0.795	0.758	0.507

Abbreviations: PINs, protein interaction networks; GC, giant component; No., number; Avg., average; h, hours. PC9R denotes the gefitinib-resistant cell line. ^1^ Small-world index > 1 indicates networks retaining small-world topology. Entropy values are normalized Shannon entropy (range 0–1). Complete properties are provided in Table S2.

**Table 3:** Genes with maximum centrality at each time point in gefitinib-sensitive PC9 and gefitinib-resistant PC9R cells.

Centrality	1 h	6 h	12 h	18 h	24 h
Sensitive PC9					
Degree	CREBBP	CDK1	EXO1	CDC6	RRM2
Betweenness	HIST1H2BJ	ABL1	MYC	PKMYT1	RRM2
Closeness	HIST1H2BJ	ABL1	MYC	PKMYT1	DTL
Eigenvector	VPRBP	CDK1	RRM2	CDC6	RRM2
Resistant PC9R					
Degree	NOP56	NOP56	CDC6	EXO1	BIRC3
Betweenness	ABL1	UBE2D1	PKMYT1	MCM10	BIRC3
Closeness	ABL1	WDR4	CDC6	MCM10	BIRC3
Eigenvector	NOP56	MRTO4	CDC6	EXO1	CASP10

At 24 h, BIRC3 dominates degree, betweenness, and closeness centrality in resistant cells, whereas CASP10 shows the highest eigenvector centrality, together suggesting late enrichment of apoptosis-regulatory nodes in the resistant network.

**Table 4:** Global properties of co-expression-augmented PINs for PC9 and PC9R cells.

Property	PC9 (Sensitive)	PC9R (Resistant)
Order (nodes)	138	116
Size (edges)	257	179
Density	0.0393	0.0376
Assortativity	0.204	0.086
Diameter	13	13
Small-world index	3.180	2.632
Degree entropy	0.816	0.783
Betweenness entropy	0.623	0.631
Closeness entropy	0.944	0.954
Eigenvector entropy	0.854	0.835
PageRank entropy	0.889	0.889
Clustering entropy	0.696	0.659

Both networks exhibit small-world topology (index > 1), positive assortativity, and low density, confirming structural equivalence at the global level.

## Data Availability

The gene expression dataset analyzed in this study is publicly available in the NCBI Gene Expression Omnibus under accession number GSE34228 at https://www.ncbi.nlm.nih.gov/geo/ (accessed on 10 September 2026). All intermediate analysis files—DEG lists, GSEA leading-edge gene sets, COXPRESdb seed lists, STRING-exported network edge files for all twelve networks, Cytoscape session files (.cys), and the Excel template used for Shannon-entropy computation—are available on request.

## Supplementary Materials

The following supporting information can be downloaded at: https://www.mdpi.com/article/10.3390/ijms27188409/s1 [56,57].

## Abbreviations

The following abbreviations are used in this manuscript:

NSCLC	Non-small cell lung cancer
EGFR	Epidermal growth factor receptor
EGFR-TKI	Epidermal growth factor receptor tyrosine kinase inhibitor
PIN	Protein interaction network
DEG	Differentially expressed gene
GC	Giant component
GSEA	Gene set enrichment analysis
IAP	Inhibitor of apoptosis
GEF	Guanine nucleotide exchange factor
EMT	Epithelial-to-mesenchymal transition
