# Supplementary material for: Time-Resolved Network Entropy Analysis Identifies a CTNND1–VAV3 Subnetwork in Gefitinib-Resistant NSCLC
Source: Int J Mol Sci. 2026 Sep 21;27(18):8409. doi: 10.3390/ijms27188409 (PMC13607449; doi:10.3390/ijms27188409)
Supplement: Supplementary file 1 [file ijms-27-08409-s001.zip › ijms-4483949-supplementary.pdf]

Supplementary Information

Time-Resolved Network Entropy Analysis Identifies a CTNND1–VAV3 Subnetwork in Gefitinib-Resistant NSCLC

Dimitra C. Tsakona1,2 and Nikolaos A. Papanikolaou3,4,5*

[1]Inter-Faculty Program on Networks and Complexity, Aristotle University of Thessaloniki, Thessaloniki 54124, Greece; [2]Department of Biology, Aristotle University of Thessaloniki, Thessaloniki 54124, Macedonia, Greece

[3]Department of Basic Sciences, Touro College of Osteopathic Medicine, Touro University, New York, NY 10027, USA

[4]Laboratory of Biological Chemistry, Aristotle University of Thessaloniki School of Medicine, Thessaloniki 54124, Macedonia, Greece; [5]Akesis Synergy Med, 49 Arkadiou Str., Thessaloniki 561521, Macedonia, Greece

*Correspondence: npapanik@touro.edu (Nikolaos A. Papanikolaou)

Contents

Supplementary Methods

Supplementary Figure S1. Volcano plots — DEG analysis, Gefitinib-sensitive PC9 cells

Supplementary Figure S2. Volcano plots — DEG analysis, Gefitinib-resistant PC9R cells

Supplementary Figure S3. Co-expression PIN comparison, PC9 vs. PC9R

Supplementary Table S1. Full network properties — Gefitinib-sensitive PC9 cells

Supplementary Table S2. Full network properties — Gefitinib-resistant PC9R cells

Supplementary Table S3. Co-expression network global properties

Supplementary Table S4. Software, database versions, access dates, and key parameter settings
Supplementary References

Supplementary Methods

SM1. Gene Expression Data Acquisition and Quality Control

Normalized gene expression microarray data were obtained from the NCBI Gene Expression Omnibus (GEO) under accession number GSE34228. The dataset comprises time-course measurements from gefitinib-sensitive PC9 and gefitinib-resistant PC9R non-small cell lung cancer cells treated with gefitinib or maintained as untreated controls at five time points: 1 h, 6 h, 12 h, 18 h, and 24 h. Sample normalization was assessed by comparing median-centered boxplot distributions across samples within each time point and cell line before differential expression analysis.

SM2. Differential Expression Analysis

Differential expression between gefitinib-treated and untreated samples was assessed with GEO2R (https://www.ncbi.nlm.nih.gov/geo/geo2r/; accessed on 16 September 2026), the NCBI web interface that executes the limma Bioconductor package on the GEO server. Default GEO2R settings were applied, including log2 transformation of expression values before model fitting and Benjamini–Hochberg false-discovery-rate adjustment of p-values. For each gene at each time point, the log2 fold change (log2FC) and adjusted p-value (adj-p) were obtained, and genes with adj-p < 0.05 were considered significantly differentially expressed. For temporal protein interaction network (PIN) construction, the top 1,000 DEGs ranked by |log2FC| per cell line per time point were retained. For co-expression-augmented PIN construction, all significant DEGs across the full 24 h treatment window were used (PC9: 19,995; PC9R: 16,776). The top-1,000 threshold was selected to generate networks of manageable and broadly comparable size and was not intended as a biologically defined cutoff.

SM3. Protein Interaction Network Construction

For each combination of cell line and time point, the corresponding top-1,000 DEG list was submitted to STRING v11 (https://string-db.org/; accessed on 16 September 2026) through the multiple-protein search interface with the organism set to Homo sapiens. Active interaction sources were restricted to experiments and databases; co-expression, text mining, gene neighborhood, gene fusion, and co-occurrence were disabled. The minimum required interaction score was left at the STRING default of medium confidence (0.400), and the resulting networks were exported in TSV format. This procedure yielded ten temporal PINs (five time points × two cell lines).

Co-expression-augmented PINs were constructed separately for each cell line. For each cell line, a 500-gene seed list was assembled from three sources: (i) the top 100 DEGs ranked by |log2FC| across the full 24 h treatment window, obtained from GEO2R as described above; (ii) 100 leading-edge genes derived from significantly enriched Hallmark gene sets (MSigDB v7.4) identified by Gene Set Enrichment Analysis (GSEA v4.1, Broad Institute desktop application) using default parameters (1,000 gene-set permutations, weighted enrichment statistic, gene-set size 15–500); and (iii) 300 EGFR co-expressed genes retrieved from COXPRESdb v7 (https://coxpresdb.jp/; accessed on 16 September 2026) using EGFR as the query protein with default settings on the Hsa-r.c2-0 reference platform. The combined 500-gene seed list for each cell line was then submitted to STRING v11 using the same interaction-source and confidence-score settings described above. Because the COXPRESdb component was seeded on EGFR, these networks were interpreted as an EGFR-centered exploratory analysis.

SM4. Network Topology and Entropy Calculations

All network analyses and visualizations were performed in Cytoscape v3.8.2. NetworkAnalyzer (built-in) was used for global topological properties, including order, size, density, number of connected components, giant-component (GC) order and size, diameter, average shortest-path length, global and local clustering coefficients (Watts–Strogatz transitivity), and degree assortativity. CytoHubba was used to rank nodes by degree, betweenness, closeness, and eigenvector centrality and to extract the top-10 betweenness-centrality (bottleneck) subnetworks with their first-order interactors. CytoNCA was used for centralization measures and cross-validation of centrality scores. Erdős–Rényi reference graphs matched on node and edge counts were generated within Cytoscape to normalize the small-world calculations.

The small-world index σ was computed as $\sigma = (C/C\_rand)/(L/L\_rand)$, where C and L are the observed clustering coefficient and average path length, and C_rand and L_rand are the corresponding values for a size-matched Erdős–Rényi random graph. Networks with $\sigma > 1$ were classified as small-world.

Five entropy metrics were computed for the temporal PINs: degree entropy, betweenness entropy, closeness entropy, eigenvector entropy, and clustering entropy. For a discrete probability distribution {p1, p2, …, pm}, with $\Sigma pi = 1$, Shannon entropy was calculated as $H = \Sigma pi \ln(pi)$ and normalized as $Hnorm = -H/\ln(m)$, yielding values between 0 and 1. For the co-expression-augmented PINs, PageRank entropy was also computed using the same Shannon-entropy normalization. Node-property distributions exported from Cytoscape were discretized before entropy calculation in Microsoft Excel. Because entropy estimates can depend on discretization choices, including bin number and bin width, no formal binning-sensitivity analysis was performed.

Degree distributions were assessed for compatibility with power-law behavior using the maximum-likelihood framework of Clauset, Shalizi, and Newman. The analysis returns the scaling exponent α, lower-bound parameter xmin, and a Kolmogorov–Smirnov goodness-of-fit p-value; $p > 0.05$ was interpreted as compatibility with a power-law tail. This test was used descriptively and does not establish a power law as the best-fitting model relative to alternative heavy-tailed distributions. The scaling exponents and goodness-of-fit p-values are provided in the associated raw data.

Supplementary Figures

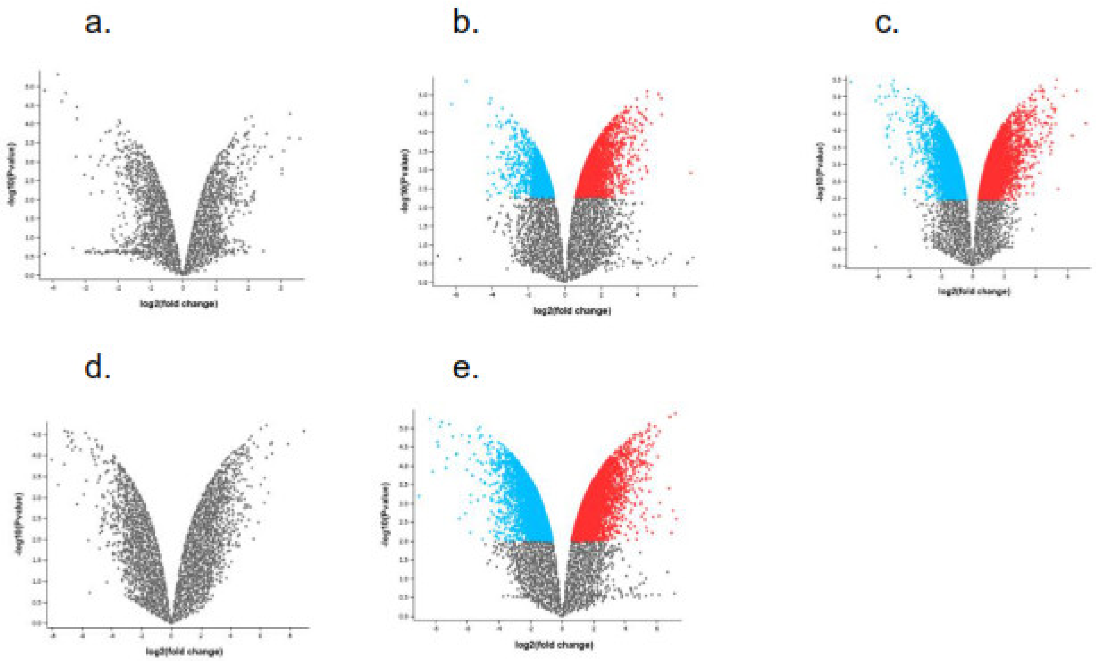

Supplementary Figure S1. Volcano plots showing differential gene expression in Gefitinib-sensitive PC9 cells at five time points (1 h, 6 h, 12 h, 18 h, 24 h) after Gefitinib administration. Each point represents one gene; red points are significantly upregulated ($log_2FC > 0$, adj-P < 0.05); blue points are significantly downregulated ($log_2FC < 0$, adj-P < 0.05); grey points are non-significant. Horizontal axis: log_2 fold change. Vertical axis: $-log_{10}$ adjusted p-value.

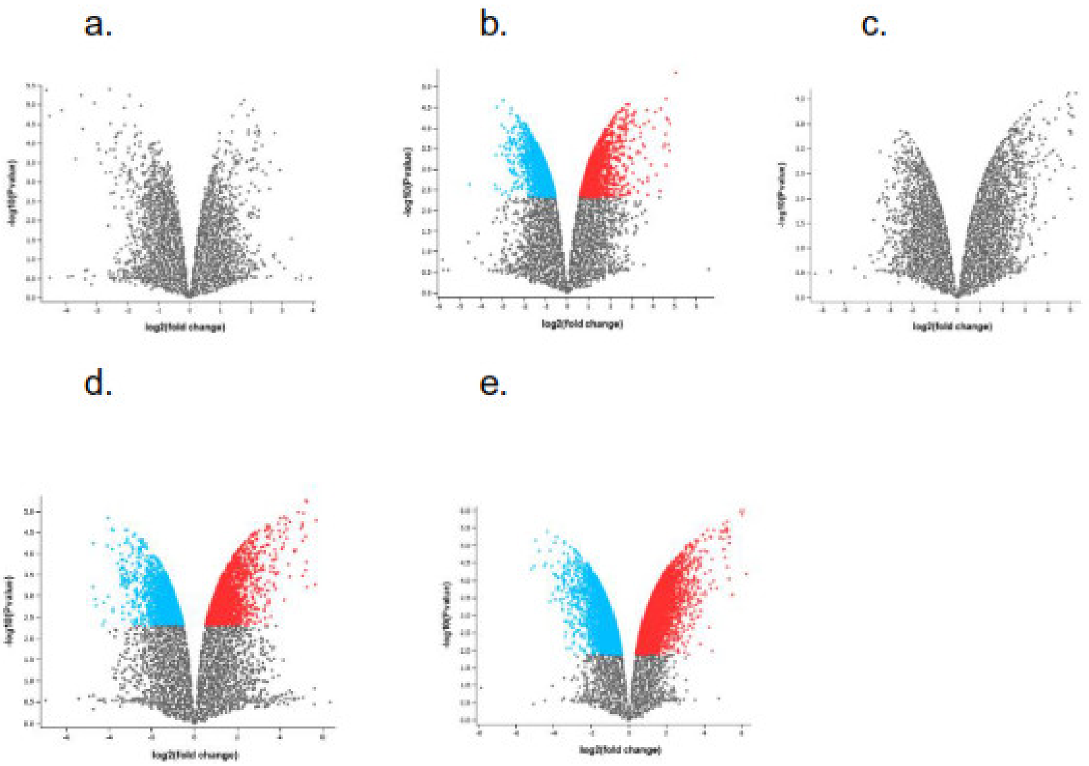

Supplementary Figure S2. Volcano plots showing differential gene expression in Gefitinib-resistant PC9R cells at five time points (1 h, 6 h, 12 h, 18 h, 24 h) after Gefitinib administration. Each point represents one gene; red points are significantly upregulated ($log_2FC > 0$, adj-P < 0.05); blue points are significantly downregulated ($log_2FC < 0$, adj-P < 0.05); grey points are non-significant. Horizontal axis: log_2 fold change. Vertical axis: $-log_{10}$ adjusted p-value.

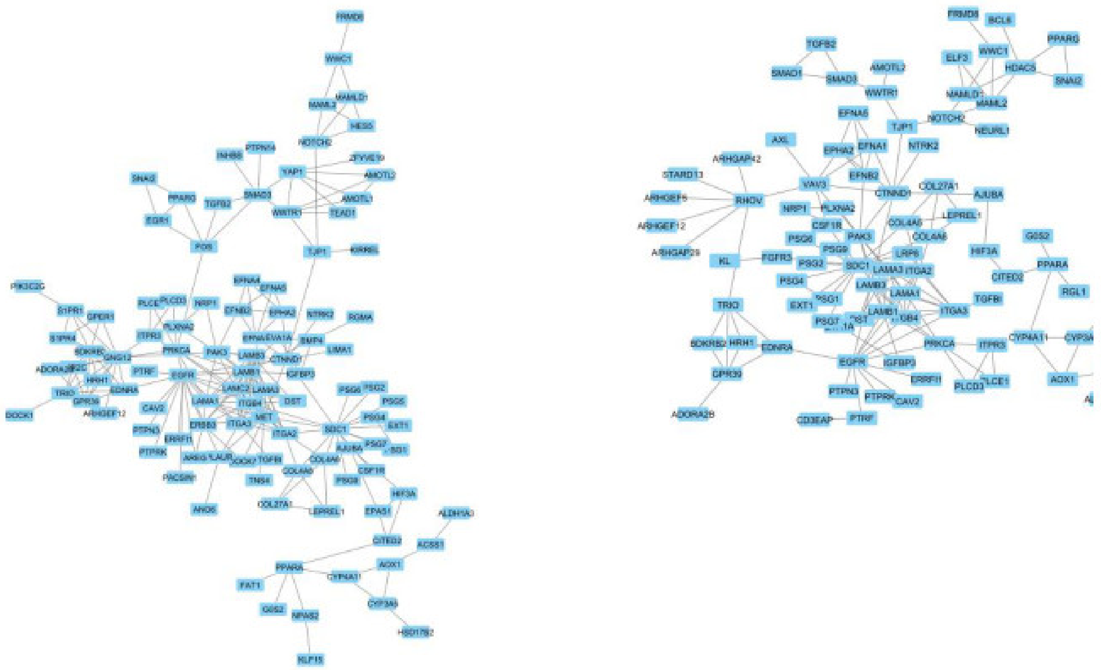

Supplementary Figure S3. Co-expression-augmented protein interaction networks for gefitinib-sensitive PC9 cells (left) and gefitinib-resistant PC9R cells (right) across the 24 h gefitinib-response window. Seed genes comprised the top 100 DEGs from GEO2R analysis, 100 leading-edge genes from significantly enriched Hallmark gene sets identified by GSEA (MSigDB v7.4), and 300 EGFR co-expressed genes retrieved from COXPRESdb v7 using the Hsa-r.c2-0 reference platform. Edges represent STRING v11 protein–protein associations supported by the experiments and databases evidence channels. Node size is proportional to degree. Despite broadly comparable global topology (small-world index: PC9 = 3.180; PC9R = 2.632; see Supplementary Table S3), the resistant network highlights high-betweenness bottleneck nodes that are not among the dominant bottlenecks in the sensitive network, including CTNND1, VAV3, HIF3A, NOTCH2, and CITED2.

Supplementary Tables

Supplementary Table S1. Complete global network properties for Gefitinib-sensitive PC9 temporal PINs at five time points

Measure	1 h	6 h	12 h	18 h	24 h
Order (nodes)	221	118	206	124	164
Size (edges)	478	140	358	150	128
No. of components	19	31	32	30	50
GC order	162	30	124	35	20
GC size	404	30	304	82	24

GC (% of total)	73%	25%	60%	28%	12%
Diameter	9	8	24	9	7
Avg. path length	4.296	3.331	8.616	3.267	2.609
ER avg. path length	3.774	4.574	4.235	4.967	7.314
Transitivity (global)	0.619	0.693	0.621	0.544	0.288
Transitivity (W&S)	0.455	0.116	0.192	0.169	0.143
Graph density	0.019	0.02	0.016	0.019	0.009
Assortativity	0.441	0.695	0.597	0.562	0.27
Degree centralization	0.107	0.222	0.141	0.353	0.152
Betweenness centralization	0.418	0.578	0.467	0.382	0.49
Closeness centralization	0.256	0.288	0.118	0.261	0.313
Small-world index	5.157	0.048	0.626	0.578	0.231
Degree entropy	0.835	0.247	0.410	0.993	0.596
Betweenness entropy	0.601	0.774	0.765	0.857	0.899
Closeness entropy	0.945	0.668	0.708	0.845	0.868
Eigenvector entropy	0.457	0.963	0.937	0.985	0.984
Clustering entropy	0.659	0.963	0.438	0.915	0.994

GC = giant component. W&S = Watts–Strogatz local clustering coefficient. ER = corresponding Erdős–Rényi random-graph value. Degree-distribution power-law exponents (α) and Kolmogorov–Smirnov goodness-of-fit p-values are included in the associated raw data. All entropy values are normalized Shannon entropy values ranging from 0 to 1.

Supplementary Table S2. Complete global network properties for Gefitinib-resistant PC9R temporal PINs at five time points

Measure	1 h	6 h	12 h	18 h	24 h
Order (nodes)	138	218	168	134	128
Size (edges)	200	543	308	158	119
No. of components	23	32	34	31	39
GC order	72	129	53	28	18
GC size	151	475	201	73	18
GC (% of total)	52%	59%	32%	21%	14%
Diameter	15	17	10	10	8
Avg. path length	6.215	6.337	3.488	2.952	2.672
ER avg. path length	4.761	3.500	4.011	5.027	3.888
Transitivity (global)	0.652	0.617	0.601	0.561	0.547
Transitivity (W&S)	0.203	0.319	0.306	0.163	0.186
Graph density	0.021	0.022	0.021	0.017	0.014
Assortativity	0.625	0.408	0.518	0.587	0.502
Degree centralization	0.186	0.227	0.368	0.311	0.066
Betweenness centralization	0.606	0.491	0.299	0.389	0.541
Closeness centralization	0.162	0.123	0.311	0.333	0.296
Small-world index	2.030	1.703	0.981	0.906	0.337
Degree entropy	0.806	0.799	0.887	0.895	0.931
Betweenness entropy	0.683	0.641	0.804	0.801	0.825
Closeness entropy	0.954	0.953	0.980	0.968	0.968
Eigenvector entropy	0.503	0.647	0.905	0.944	0.987
Clustering entropy	0.551	0.714	0.795	0.758	0.507

See Supplementary Table S1 for notation.

Supplementary Table S3. Global properties of co-expression-augmented PINs for Gefitinib-sensitive PC9 and resistant PC9R cells

Property	PC9 (Sensitive)	PC9R (Resistant)
Order (nodes)	138	116
Size (edges)	257	179
Density	0.0393	0.0376
Assortativity	0.204	0.086
Diameter	13	13
Small-world index	3.180	2.632
Degree entropy	0.816	0.783
Betweenness entropy	0.623	0.631
Closeness entropy	0.944	0.954
Eigenvector entropy	0.854	0.835
PageRank entropy	0.889	0.889
Clustering entropy	0.696	0.659

Both networks retain small-world properties (index > 1). Eigenvector entropy is modestly higher in PC9 sensitive cells (0.854 vs. 0.835), in contrast to the resistant-higher pattern observed in the temporal PINs at 1 h.

Supplementary Table S4. Software, database versions, access dates, and key parameter settings

**Resource / software**	**Version**	**Access date**	**Purpose**	**Key settings**
NCBI GEO / GSE34228	GSE34228	16 September 2026	Source microarray dataset	PC9 and PC9R; 1, 6, 12, 18, and 24 h; treated vs. untreated controls
GEO2R	Web interface	16 September 2026	Differential-expression analysis	Default GEO2R settings; log2 transformation; Benjamini–Hochberg adjustment; adj-p < 0.05
STRING	v11	16 September 2026	Temporal and co-expression-augmented PIN construction	Homo sapiens; experiments + databases only; medium confidence 0.400; other evidence channels disabled
GSEA	v4.1	N/A	Hallmark gene-set enrichment	1,000 gene-set permutations; weighted statistic; gene-set size 15–500
MSigDB	v7.4	N/A	Hallmark gene-set source	Hallmark collection
COXPRESdb	v7	16 September 2026	EGFR co-expression seed genes	EGFR query; default settings; Hsa-r.c2-0 reference platform; 300 genes
Cytoscape	v3.8.2	N/A	Network analysis and visualization	Used for all network analyses and visualizations
NetworkAnalyzer	Built-in	N/A	Global topological properties	Order, size, density, components, GC, diameter, path length, clustering, assortativity
CytoHubba	Cytoscape plugin	N/A	Centrality ranking and bottleneck subnetworks	Degree, betweenness, closeness, eigenvector centrality; top-10 bottlenecks + first-order interactors
CytoNCA	Cytoscape plugin	N/A	Centralization and score cross-validation	Centralization measures; cross-validation of centrality scores
Microsoft Excel	Not specified	N/A	Entropy calculation	Discretization of exported node-property distributions; normalized Shannon entropy

N/A, not applicable for locally installed desktop software or plugins. Web-resource access dates match those reported in the main manuscript.
